# Transmission patterns of *Leishmania tropica* around the Mediterranean basin: Could Morocco be impacted by a zoonotic spillover?

**DOI:** 10.1371/journal.pntd.0010009

**Published:** 2022-01-13

**Authors:** Imane El Idrissi Saik, Chaimaa Benlabsir, Hassan Fellah, Meryem Lemrani, Myriam Riyad

**Affiliations:** 1 Laboratory of Cellular and Molecular Pathology, Research Team on Immunopathology of Infectious and Systemic Diseases, Faculty of Medicine and Pharmacy, Hassan II University of Casablanca, Casablanca, Morocco; 2 Laboratory of Parasitology and Vector-Borne-Diseases, Institut Pasteur du Maroc, Casablanca, Morocco; Insitut Pasteur de Tunis, TUNISIA

## Abstract

Cutaneous leishmaniasis (CL) due to *Leishmania tropica* is a neglected tropical disease characterized by a wide geographical distribution in the Mediterranean basin and is endemic in several of its countries. In addition, the vector *Phlebotomus sergenti* is abundantly present all around the basin. Its transmission cycle is still subject to debate. In some countries, the presence of an animal reservoir has been confirmed. In Morocco, CL due to *L*. *tropica* has risen since the 1980s and has spread widely to become the most abundant form of leishmaniasis in the territory. However, the anthroponotic transmission is so far the only recognized mode, despite recordings of *L*. *tropica* infection in animal hosts. In this review article, we assess the situation of CL due to *L*. *tropica* in the Mediterranean basin with a focus on Morocco and gather knowledge about any potential zoonotic transmission in the country. A concomitant zoonotic transmission could explain the persistence of the disease in areas where human protective measures combined with vector management did not help reduce the disease burden.

## Author summary

Cutaneous leishmaniasis (CL) due to *Leishmania tropica* is a neglected tropical disease still considered to be anthroponotic in Morocco despite evidence of zoonotic transmission in other endemic countries of the Mediterranean basin. Its high incidence in the country makes it necessary to investigate whether or not a potential concomitant zoonotic transmission exists alongside the anthroponotic one. Moreover, several reports of natural animal infection have been noted in the country. In this paper, we review how CL due to *L*. *tropica* is present around the Mediterranean basin, with a particular focus on the Moroccan foci, and try to unveil the existence of a zoonotic transmission in the area. A deep understanding of the transmission patterns of *L*. *tropica* in the region is necessary for the implementation of effective control measures and a better assessment of the disease burden. In addition, the complexity of this neglected disease requires more fundamental and applied research to unveil potential factors involved in its spread.

## Introduction

The development and spread of infectious diseases are influenced by humans, animals, and the environment, and around 61% of human infections are zoonotic. Zoonotic diseases are constantly emerging and expanding, and new transmission patterns are being uncovered [1]. Leishmaniasis is a neglected tropical parasitic disease caused by intracellular protozoa of the genus *Leishmania* and transmitted through the bite of female sand flies. The disease is distributed worldwide and characterized by 3 main clinical forms: cutaneous leishmaniasis (CL), visceral leishmaniasis (VL), and mucocutaneous leishmaniasis (MCL) [2]. In the world, 0.7 to 1 million new CL cases are reported annually to WHO from 86 CL endemic countries [3]. In the Old World, *Leishmania major*, *Leishmania aethiopica*, and *Leishmania infantum* are responsible for zoonotic CL (although *L*. *infantum* is also mainly the cause of zoonotic VL). *Leishmania tropica* is mainly considered responsible for anthroponotic CL. Healed CL due to *L*. *tropica* can sometimes be followed by *Leishmaniasis recidivans* (LR), usually appearing as new lesions around the primary healed ones and characterized by a progressive expansion [2].

Around the Mediterranean basin, *L*. *tropica* has a wide geographical distribution in Greece, Turkey, and North African countries. CL due to *L*. *tropica* is characterized by its highest incidence in Morocco where it has been expanding since the 1990s [4]. These last decades, the geopolitical situation and wars in some east Mediterranean countries also led to a rise in CL due to *L*. *tropica* cases [5]. The emergence or reemergence of the disease is under the control of several factors, mainly environmental, which are crucial in initiating the spread of the parasite among the human population, and socioeconomic factors, which potentially impact the morbidity [6]. As climate changes and human mobility expands, the prevalence of CL due to *L*. *tropica* could rise without the involvement of an anthropogenic spread [7]. The anthroponotic character of *L*. *tropica* is still debated. Several potential host species are now considered reservoirs in Algeria, Tunisia, Palestine, and Israel [8–10]. However, in Morocco, despite the parasite being identified in a number of animal hosts, it is still considered as anthroponotic. This review article aims to gather existing data about transmission patterns of CL due to *L*. *tropica* around the Mediterranean basin and Morocco and identify research gaps that need to be addressed.

## Methodology

A review of the literature was conducted through articles obtained via PubMed, Web of Science, and Google Scholar databases. Several sets of keywords were used for this search. The terms “Cutaneous leishmaniasis” and “*Leishmania tropica*” were combined to the following keywords: “Zoonotic,” “Reservoir,” “Animal host,” “Mediterranean,” “Morocco,” “Vector,” “Epidemiology,” and “Distribution.” Articles in English and French were browsed. No year limitation was assigned. Several combinations of these keywords were entered, and articles were selected from titles and abstract matching the reviews’ aim. Relevant articles on the epidemiology of CL due to *L*. *tropica* in the Mediterranean basin, the distribution of *L*. *tropica*’s vector, and the animal hosts of *L*. *tropica* in the same area were included in the manuscript.

### Geographical distribution of cutaneous leishmaniasis caused by *Leishmania tropica* in the Mediterranean basin

CL due to *L*. *tropica* has widely been reported across the Mediterranean basin. Syria is historically endemic to CL due to *L*. *tropica* for centuries and had previously the highest burden for this form of leishmaniasis in the region [11]. These last decades, political instability and wars led to an increase in cases. Even though the disease is mainly prevalent in Aleppo, some governorates have recorded unprecedented numbers of cases after the war started. This situation led to the displacement of a huge number of refugees to neighboring countries, ultimately leading to outbreaks of CL in refugee camps [5,11]. Two Mediterranean neighbors of Syria, Turkey and Lebanon, observed a sudden increase of CL cases and experienced outbreaks of CL in refugee camps. Prior to the Syrian civil war, the peak of CL cases recorded in Lebanon was only 6 cases per year, but since the arrival of refugees, an unprecedent increase in cases was recorded. In 2012, a CL outbreak in refugee camps in Lebanon’s borders was 85% due to *L*. *tropica* [12,13]. A similar situation was observed in refugee camps of Turkey, although Turkey already accounts as one of the main foci of CL due to *L*. *tropica* in the region. The largest portion of cases originates from the Mediterranean southern coast of Turkey [11]. In Palestine and Israel, CL due to *L*. *tropica* is endemic, although its cycle of transmission is not fully elucidated in the region, and in Egypt, the first *L*. *tropica* CL cases were reported in 2009 from a classical *L*. *major* focus in North Sinai [14].

In North Africa, Morocco has the highest burden of this form of CL. Currently, CL due to *L*. *tropica* is characterized by the widest geographic distribution in the country, as 43% of CL cases (54,838 cases of CL notified in total) were due to *L*. *tropica* between 2008 and 2017 [15]. It is present in an area extending from the Atlantic Ocean all along the Atlas Mountains and close to the Mediterranean Sea [16]. During the 1980s, this form prevailed in scattered hypoendemic rural foci and small towns located in the arid mountains where the disease was sporadic. The first reported case dates back to 1989 in Tanant located in the province of Azilal [17]. Since then, *L*. *tropica* cases have increasingly been reported from different regions of the country. In 1991, an ecoepidemiological study unveiled a vast *L*. *tropica* focus in central and southern areas of the country such as Guelmim, Agadir, and Essaouira [18]. In 1996, an urban epidemic was reported in Taza Province [19]. In the early 2000s, outbreaks were growingly notified from various central and northern Moroccan regions, such as Zouagha Moulay Yaacoub (Province of Fes) and Chichaoua, an epidemic focus near Marrakesh [20,21]. In 2006, *L*. *tropica* even spread to Settat Province that was nonendemic to CL. From 2007 to 2012, 553 cases were reported from this province, most of which originated from the same locality (Lbrouj) [22]. More recently, *L*. *tropica*’s presence was confirmed in semirural localities of Toundout and Agdz, which are separated by the Atlas Mountains, north and south of Ouarzazate. This was the first report of *L*. *tropica* in the south of the Atlas Mountains, since the species is endemic to central arid and semiarid regions and northwest to the Atlas Mountains [23]. Over the last decades, *L*. *tropica* foci have spread to several regions of Morocco including those that previously reported CL caused by *L*. *major* or *L*. *infantum*. Recently, a survey carried out in Casablanca highlighted the possible introduction of *L*. *tropica* to urban areas [4].

In Algeria and Libya, only sporadic cases of *L*. *tropica* infection were notified [24]. Nevertheless, cases of CL due to *Leishmania killicki* (syn. *L*. *tropica*) are continuously reported in Algeria, Libya, and Tunisia [25]. It has been identified for the first time in 1980 in southeastern Tunisia, then in 2006 in Libya, and 2009 in Algeria. Since then, sporadic cases were identified in center, southwestern, and northern Tunisia, as well as in northern part of the Algerian Sahara and in northeastern Algeria [24].

Finally, in southern Europe, CL due to *L*. *tropica* has only been reported in Greece, where it was recorded for the first time in 1984 and occurs sporadically. Nevertheless, the proximity of certain European countries to endemic neighbors alongside the anthroponotic character generally recognized for *L*. *tropica*, as well as the presence of the vector *Phlebotomus sergenti* poses a major risk of introduction of CL due to *L*. *tropica* in south Europe [26].

### *L*. *tropica*’s vector distribution

*Phlebotomus (Paraphlebotomus) sergenti* is the proven main vector of *L*. *tropica*. It is characterized by a wide geographical distribution in the Mediterranean basin [27,28]. It is a proven vector of *L*. *tropica* in Algeria, Israel, Morocco, and Tunisia. Moreover, it is suspected to be a vector in each of Greece, Libya, Palestine, Syria, and Turkey [29].

Even though *P*. *sergenti* is still the main proven vector of *L*. *tropica*, *Phlebotomus arabicus* and *Phlebotomus similis* have also been studied for their vectorial capacities for *L*. *tropica*. Indeed, *P*. *arabicus* has been proven as a potential vector in northern Israel, and the widespread presence of *P*. *similis* (classified in the same subgenus as *P*. *sergenti*) in the island of Crete makes it a likely vector of *L*. *tropica* in the region [30]. Moreover, in Greek refugee camps, *L*. *tropica* was detected in other species of *Phlebotomus* sand flies, mainly *Phlebotomus tobbi* and *Phlebotomus perfiliewi* that could be potential vectors of *L*. *tropica* [31]. Although other *Leishmania* species may be isolated from *P*. *sergenti* [32], it is still considered as a specific vector able to only mature *L*. *tropica* in experimental conditions [33]. However, for a sand fly to be incriminated as a vector of human leishmaniasis, its anthropophilic behavior as well as its infection with the *Leishmania* parasite originating from the same area must be proven [34].

Interestingly, the geographic distribution of *P*. *sergenti* is wider than the parasite it harbors. This species is distributed in CL endemic and nonendemic Mediterranean countries, where it is widely spread across semiarid regions. In Morocco, the first detection of *L*. *tropica* in *P*. *sergenti* was reported in 1991 from the first documented focus of Tanant [35]. Since then, *P*. *sergenti* infection with *L*. *tropica* was reported in central and the northern parts of the country [36–38]. In Morocco, the distribution of sand flies is mainly determined according to the altitude [39]. *P*. *sergenti* and *Phlebotomus papatasi* are the most prevalent sand fly species, and they equally share the territory, although their abundance and density are under the influence of ecological factors. For instance, *P*. *sergenti*’s density is significant in arid and Saharan zones, whereas *P*. *papatasi* is capable of well adapting to different arid zones [40]. In contrast with *P*. *papatasi*, *P*. *sergenti* was found up to 1,400-m altitude in Al-Haouz region of the High Atlas mountains [39]. In this region, *P*. *sergenti* was distinguished by an anthropophilic character and was mainly collected in domestic and peridomestic habitats [41].

Although *P*. *sergenti* was previously commonly found in mild to high altitudes and was described as a “mountainous” species, in Morocco, it was found in areas up to 2,000 m [42]. In turn, it was mostly found between 0 and 200 m in southern Anatolia, showing a capacity of adaptation in lower altitudes where usually a more dense human population lives [43].

Interestingly, while no autochthonous cases of CL due to *L*. *tropica* have been recorded in Europe, *P*. *sergenti* is present, and even high infection rates of *L*. *tropica* were detected in 21 sand fly species in Greek refugee camps [31]. This species was captured in south and central regions of Portugal [44], identified in the south of France and Corsica, in Spain mainland, the Canary Islands, the Balearic Islands, and Sicily [33], as well as in Cyprus [45]. Its presence has even been notified in southeastern parts of Romania [46]. Moreover, the high density of *P*. *sergenti* in the Iberian Peninsula and the identification of one common mitochondrial lineage in both Morocco and southwestern Europe, in addition to the presence of *L*. *tropica* in northern parts of Morocco [47], as well as the bioclimatic affinity between northern Morocco and southeast of the Iberian Peninsula, raise the question of the possible introduction of *L*. *tropica* [33]. Southern Europe is similar to northern Morocco in terms of climate and ecology, and the widespread presence of *P*. *sergenti* poses a risk of introduction of *L*. *tropica* to the region. Even though sand flies are known to travel short distances, the anthroponotic character of *L*. *tropica* poses a threat for the emergence of cases, since a heavy traffic of potentially infected tourists or migrants exists between this region and the endemic North African foci.

### Mammalian hosts of *L*. *tropica*

**Fig 1:**
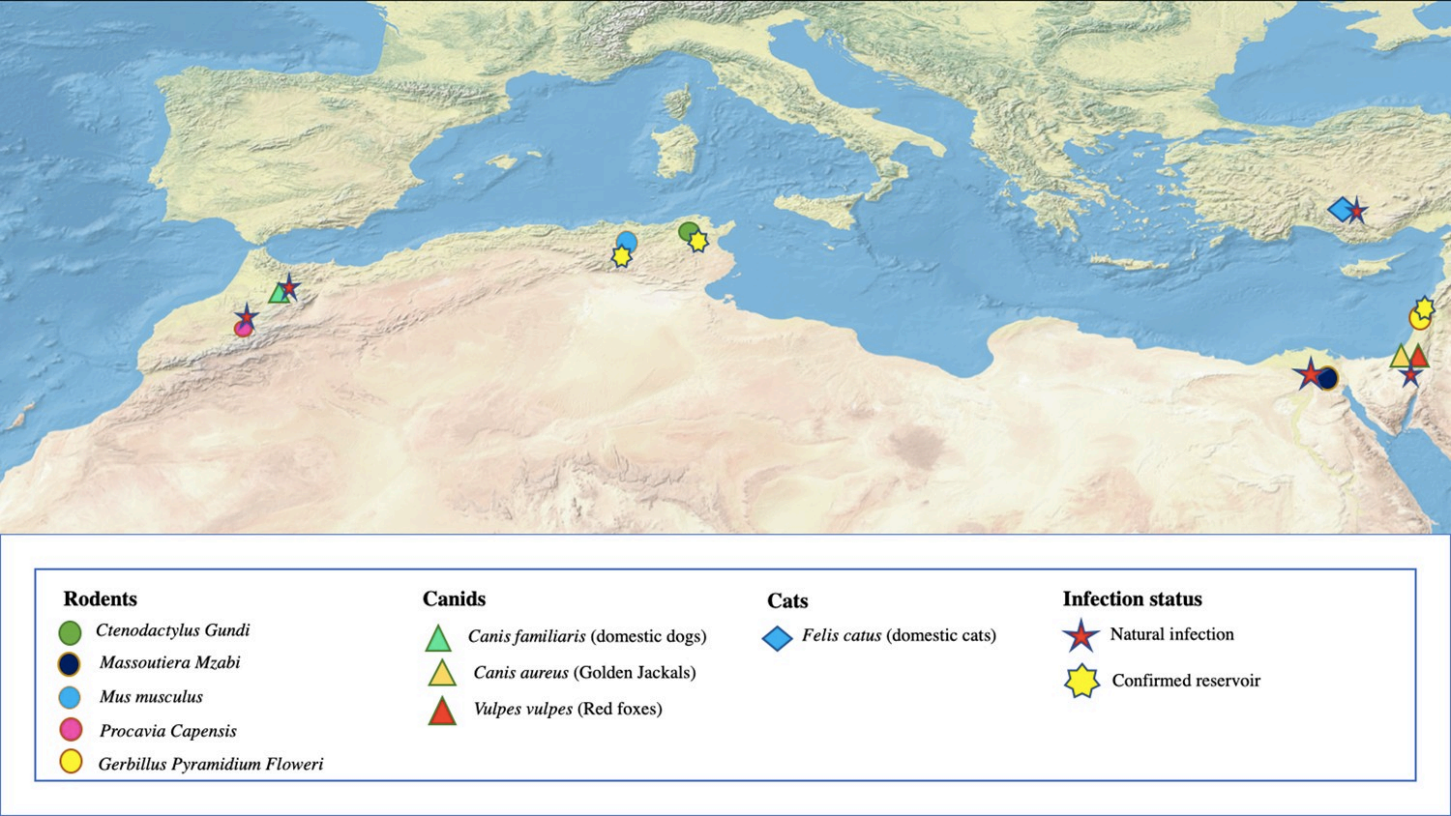
Distribution and infection status of mammalian hosts of *L*. *tropica* in the Mediterranean basin. Reported mammalian species infected with *L*. *tropica* (rodents, canids, and cats) are displayed alongside the reservoir status (confirmed or not). *Image source*: *Made with Natural Earth* (https://www.naturalearthdata.com/downloads/10m-natural-earth-1/10m-natural-earth-1-with-shaded-relief-and-water).

The anthroponotic status of CL due to *L*. *tropica* is disputed. Some potential mammalian hosts have been investigated and could contribute to the transmission of the disease (Fig 1). In Morocco, dogs were found to be infected by *L*. *tropica* and presented cutaneous lesions as well as visceral manifestations [48–50]. Despite that, dogs in Morocco are considered as accidental hosts, and the cycle of transmission of *L*. *tropica* is still to this date considered to be anthroponotic. However, the transmission cycle in the country is not fully elucidated. Some sporadic cases are reported from rural areas where the anthroponotic transmission alone is not sufficient to explain their occurrence [16]. For instance, in Imintanout (Chichaoua Province), high numbers of CL cases are still being recorded despite the efforts deployed by the Ministry of Health since the first outbreak in 2000, hinting that the persistence of the parasite among the population could be not only due to an anthroponotic transmission in this rural and lightly populated region, but also to the existence of a potential animal reservoir [51]. Rodents were identified as potential hosts in some Moroccan *L*. *tropica* foci. In the Azilal focus, *P*. *sergenti*’s blood feeding sources where identified: 7 female *P*. *sergenti* were found to feed on rodents, and 1 was infected with *L*. *tropica* [37]. Moreover, *L*. *tropica* DNA was recently detected in *Mus musculus* from central Morocco [52].

In other North African countries, CL cases due to *L*. *tropica* are lower. This coupled to their spatial distribution makes it impossible to deduce an anthroponotic transmission [16]. In Tunisia and Algeria, respectively, Gundi (*Ctenodactylus gundi*) and *Massoutiera mzabi* were suggested as possible reservoir hosts of the *L*. *killicki* (syn. *tropica*) [8,9]. Interestingly, the same *L*. *tropica* genotype was detected in Gundis, humans, and *P*. *sergenti* within the same southeastern Tunisian *L*. *tropica* foci [9]. Moreover, in Egypt, a wild-caught rodent, *Gerbillus pyramidum floweri* was found to be infected by *L*. *tropica* [14].

Throughout the Mediterranean basin, several rodent species were investigated for their capacity to be *L*. *tropica* hosts. No apparent lesion was detected in black rats (*Rattus rattus*) experimentally infected with *L*. *tropica*, making them a potential asymptomatic host for the parasite. Moreover, these black rats originate from an endemic *L*. *tropica* focus in Turkey and were found to be infective for the sand fly vector [53]. This implies that they could easily circulate the parasite while being asymptomatic. More recently, natural infection with *L*. *tropica* was reported from small wild rodents of Turkey [54]. In Israel, Rock hyraxes (*Procavia capensis*) are now considered as a reservoir [55]. Higher prevalence of the disease was linked to the expansion of hyrax populations in peri-urban areas [56]. The reservoir status of hyraxes was confirmed experimentally through demonstrating its cyclical transmission by sand flies [57]. These rodent species are widely distributed in Asia and Africa. In the same region, other domestic and wild animals were found to be carriers of *L*. *tropica*. Wild canids such as golden jackals and red foxes were infected by *L*. *tropica* and thus may transmit this species from infected hyraxes to naive hyrax populations or even humans [58]. In addition, *L*. *tropica* was detected in cats in Turkey and in wild rodents and bats in Ethiopia [59–61], and a dog presenting mucocutaneous lesions due to *L*. *tropica* was notified from Jerusalem [62]. Therefore, the risk of a spillover from any of these potential animal reservoirs to humans cannot be excluded, and the disease could become zoonotic in favorable conditions.

## General discussion

**Fig 2:**
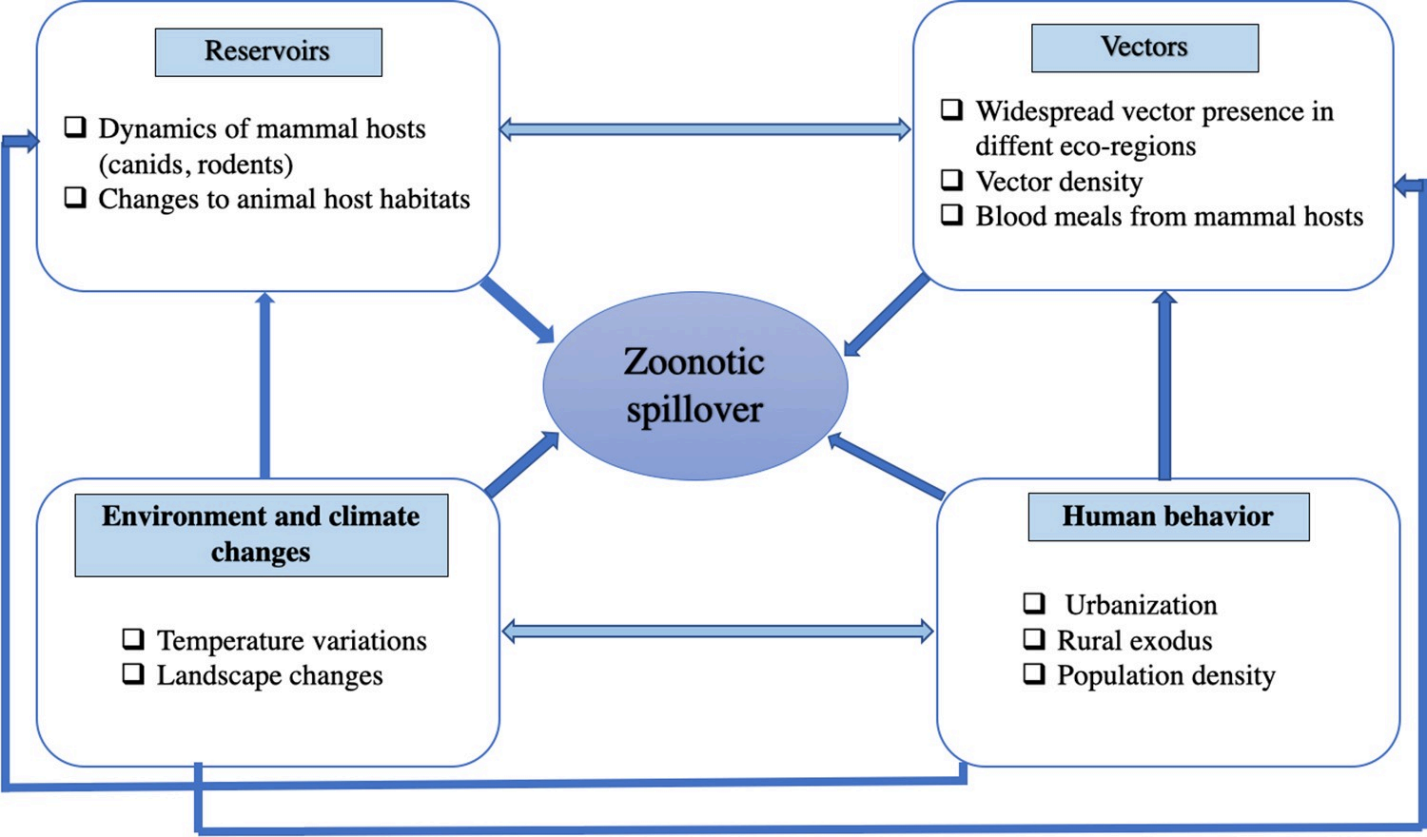
Network of factors that could lead to a zoonotic spillover of *L*. *tropica* from one of the potential mammalian hosts in Morocco.

As mentioned earlier, few mammalian species, such as rodents, canids, and cats, were found to be hosts of *L*. *tropica* in the Mediterranean basin. Rock hyraxes are now considered as reservoirs in Palestine and Israel where both anthroponotic and zoonotic transmission cycles coexist. In Morocco, the relative variety of animal hosts that were shown to be infected by *L*. *tropica* suggests that its transmission cycle may also be zoonotic (Fig 2). Since 60% of emerging human diseases have an animal origin, a deep understanding of the intertwined relationship between mammalian host distribution, pathogen repartition, and human disease pattern is essential [63,64]. For instance, a potential zoonotic cycle for *Leishmania donovani* in the Indian subcontinent was raised since nonhuman mammalian hosts were found to be infected by this predominantly anthroponotic species [65].

Although *P*. *sergenti* is still the main vector of *L*. *tropica*, and its geographic distribution is wider than the parasite it harbors, *L*. *tropica* has a wider range than expected of other potential permissive sand fly species. For instance, in Israel, *P*. *sergenti* and *P*. *arabicus* are both pillars of the transmission cycle of *L*. *tropica*: *P*. *sergenti* harbors *L*. *tropica* in the southern focus whereas *P*. *arabicus* spreads it in the northern focus. Rock hyraxes are recognized as reservoir hosts in both foci [66]. In Morocco, *P*. *sergenti* was also found to be present in very diverse ecological settings, showing a capacity of adaptation to different biotopes [42]. The direct impact of environmental and climate change on the spread of vector borne diseases has been widely demonstrated. Modifications in climatic factors induce changes in spatiotemporal distribution of vector borne diseases, whether it is by impacting survival and reproduction of the vector, parasite life cycle, or host movements [67,68]. An increase in temperature could extend the activity period of the vectors, eventually leading to a higher transmission of the disease rather than an increase in density of the vector [69]. In a recent focus of central Morocco, the exposure to *P*. *sergenti* assessed by the difference of the vector density was the only factor associated with CL due to *L*. *tropica* [70]. Therefore, it appears that surveilling the presence of *P*. *sergenti* is a key measure to controlling the spread of *L*. *tropica* among the population. To control adult phlebotomine sand flies, a wide range of techniques have been used, mostly involving insecticides. In areas where *P*. *sergenti* was the dominant vector, a noticeable reduction in CL incidence was observed following the introduction of Insecticide treated nets. However, it seemed to be less effective in Morocco, mainly due to lack of compliance [70,71]. Moreover, the identification of pathogen DNA in its arthropod vector—Xenomonitoring—can be used to infer the presence of a pathogen in an area. This less invasive option could be implemented as a first warning method to prompt further monitoring, treatment, or vector control. In addition, a continuous entomological surveillance of vector distribution and abundance should be performed and regularly updated, not only in endemic areas, but also in areas at risk of emergence throughout the Mediterranean basin.

Environmental changes to habitats of hosts and vectors affect the potential manifestation of zoonotic diseases. In Israel, *P*. *sergenti* was found to have an affinity for natural rocky habitats (rock crevices and caves) as well as artificial rocky habitats (rock piles). This affinity could be explained by the fact that rock hyraxes live in these rocky habitats. *P*. *arabicus*, incriminated in *L*. *tropica* transmission, also shares the same habitat preferences as *P*. *sergenti*. Anthropogenic landscape changes, particularly the increase of rock piles, resulted in an increase of *L*. *tropica* CL cases in the region [56].

Furthermore, human factors play a pivotal part in the expansion of the disease. For instance, unbounded urbanization greatly influences the number of CL cases and contributes to its maintenance [72]. Wars and conflicts can also influence CL prevalence, as well as impair reporting systems and control programs [5]. Moreover, traveling to endemic areas and rural exodus could lead to the introduction of CL due to *L*. *tropica* in urbanized settings [4]. In Morocco, a recent paper suggests that the spread of new CL due to *L*. *tropica* foci is linked to human travel, since the country’s road and railway infrastructure system has evolved in the recent years, increasing population movements [73]. The diagram displayed in Fig 2 shows the intricacy of a network of multiple factors that could favor a zoonotic spillover from animal hosts of *L*. *tropica* in Morocco (Fig 2).

On the other hand, adequate and quick diagnoses are key as it will allow a better and nondelayed patients’ management reducing the size of the human population’s reservoir for the vector. Unfortunately, it still mainly relies on the use of invasive sampling processes. Thus, researching and testing harmless and accurate sampling techniques requiring little conservation measures are crucial in low socioeconomic settings, particularly in remote endemic areas. Smears are used in field settings where skin biopsies and aspirates are more difficult to perform. A systematic review and meta-analysis showed that the highest estimates for sensitivity and specificity were found in tests performed from smears samples, and they appeared to be as efficient in detecting *Leishmania* infection as more invasive procedures [74]. The accuracy of swabbing, another patient-friendly technique, was assessed in an endemic area of Morocco. The molecular detection of *Leishmania* from swab samples was less sensitive than the traditional techniques, and this could be due to the low parasitic load obtained from swab samples [75]. Moreover, to assess *Leishmania* infection, patients urine was also found to be useful as a diagnostic tool in the surveillance of CL and VL [76].

In addition to these noninvasive sampling methods, further efforts must be taken into developing accurate, cost-effective, and easily performed tests in remote endemic areas. Rapid diagnosis tests (RDTs) can offer an accurate and user-friendly alternative for remote areas, as they can instantly give results while the patient is still in consultation. A recent study conducted in Morocco compared the efficacy of an RDT to standard tests to diagnose patients from various endemic areas. Even though the test had a relatively low sensitivity, it still could be used in isolated localities and would be an important addition to clinical CL management in Morocco. However, further improvements need to be done in terms of cost [77].

## Concluding remarks

The complexity of *L*. *tropica*’s transmission patterns in the Mediterranean context highlights the need of a multidisciplinary approach to control the disease. The current Coronavirus Disease 2019 (COVID-19) pandemic spotlights the threat for humans posed by potential outbreaks of zoonotic infectious diseases, making it necessary to watch closely emerging infectious diseases and fully understand the risk factors involved in their spread. In Morocco, a zoonotic spillover from one of the potential animal hosts of *L*. *tropica* is very likely, and it may enhance the disease’s spread or may even impact the clinical manifestations, leading to more severe ones. Some gaps are still hampering the fight against CL due to *L*. *tropica* in our country. For instance, the reporting system still needs improvements, especially in remote endemic areas. Moreover, not enough investigations were done regarding a potential animal reservoir, reducing the efficiency of the preventive measures taken by health instances. This added to a gap in applying protective measures in endemic localities by local populations seriously affects the disease burden. Finally, the general public is still not entirely aware of the disease, making it urgent to instigate awareness campaigns throughout the country.

Therefore, the existence of concomitant anthroponotic and zoonotic transmission cycles should not be overlooked. If a zoonotic transmission cycle is proven, a targeted control strategy of the animal reservoirs should be set up. It is therefore important to closely monitor the evolution of CL due to *L*. *tropica* in Morocco, to collect as much data as possible from endemic areas to understand its transmission pattern. It is also important to consider the training of professionals in endemic remote areas in order to improve the health reporting system. Moreover, a diverse group of experts should be involved in decision-making as part of One Health collaboration such as epidemiologists, physicians, entomologists, veterinarians, and researchers. The complexity of Leishmaniases and infectious diseases in general recalls for integrating a multidisciplinary approach to control their spread. In the case of CL due to *L*. *tropica*, this global approach could lead to not only better apprehend the disease dynamics, but also initiate the establishment of more efficient preventive techniques that take into consideration every aspect of the disease. To promote a coordinated response to zoonotic public health challenges, efficient One Health actions should take into consideration 3 types of factors: (i) individual factors such as education and training; (ii) organizational factors including communication and organizational structures; and (iii) network factors including network structures, leadership, and management. The implementation of these factors is crucial to fostering effective collaborations and multisectorial response to health concerns [78]. The first goal of this One Health collaboration should be the improvement of communication between the different sectors involved as well as the communication surrounding the disease to raise the public’s awareness. Therefore, a crucial step is the establishment of multisectorial committees that could guide operations in case of zoonotic outbreaks.

Nonendemic Mediterranean countries must be prepared for the next potential CL outbreak. Thus, it is urgent to prioritize research in basic and applied science not only to complete the knowledge of the transmission cycles due to *L*. *tropica*, but also to design new diagnostic and prevention tools. When implementing control programs, local sand fly epidemiology and behavior and multiple local transmission pathways must be taken into consideration. Finally, local populations should play an active role in the control process, and the control methods used must be sustainable.

**Key Learning Points**

- Cutaneous leishmaniasis (CL) due to *Leishmania tropica* is endemic to many countries of the Mediterranean basin where it is widely distributed.
- Several mammalian hosts were found to be naturally infected by *L*. *tropica* in the Mediterranean basin, and some were incriminated as reservoirs.
- *Phlebotomus sergenti* is present in nonendemic north Mediterranean regions, suggesting the possible introduction of *L*. *tropica* in these areas.
- A potential concomitant zoonotic transmission in Morocco should not be overlooked.
- One Health initiatives are key to implementing efficient control programs.

**Top Five Papers**

1. Burza S, Croft SL, Boelaert M. Leishmaniasis. Lancet. 2018;392:951–970. doi: 10.1016/S0140-6736(18)31204-2
2. Chaara D, Haouas N, Dedet JP, Babba H, Pratlong F. Leishmaniases in Maghreb: An endemic neglected disease. Acta Trop. 2014;132:80–93. doi: 10.1016/j.actatropica.2013.12.018
3. Aoun K, Bouratbine A. Cutaneous Leishmaniasis in North Africa: a review. Parasite. 2014;21:14. doi: 10.1051/parasite/2014014
4. Han BA, Kramer AM, Drake JM. Global Patterns of Zoonotic Disease in Mammals. Trends Parasitol. 2016;32:565–577. doi: 10.1016/j.pt.2016.04.007
5. Ready PD. Leishmaniasis emergence in Europe. Euro Surveill. 2010;15(10):29–39. doi: 10.2807/ese.15.10.19505-en
